# Challenges in the management of bone metastases: focus on differentiated thyroid cancer and neuroendocrine tumors

**DOI:** 10.1210/jendso/bvag065

**Published:** 2026-03-25

**Authors:** Julie Chen, Joy Y Wu

**Affiliations:** Department of Medicine, Division of Endocrinology, Stanford University School of Medicine, Stanford, CA 94305, USA; Department of Medicine, Division of Endocrinology, Stanford University School of Medicine, Stanford, CA 94305, USA

**Keywords:** thyroid cancer, neuroendocrine tumors, bone metastases, skeletal-related events, antiresorptive, bisphosphonate

## Abstract

Bone metastases represent an uncommon but clinically significant complication in endocrine malignancies including differentiated thyroid cancer and neuroendocrine tumors. Although these malignancies typically follow an indolent course, the development of bone metastases is associated with a worse prognosis and increased risk of skeletal-related events. The optimal strategies for the prevention and management of bone metastases in these patients remain unclear and are largely extrapolated from studies in other solid tumors. This presents a unique clinical challenge as treatment decisions need to balance the prevention of skeletal-related events against the potential risks of prolonged antiresorptive therapy in patients expected to have a long survival time. This article reviews the clinical complexities involved in managing bone metastases in differentiated thyroid cancer and neuroendocrine tumors. The clinical cases presented highlight key considerations to guide clinical decision-making when treating these specific patient populations.

Bone metastases are frequently associated with skeletal related events (SREs), including pathological fractures, bone pain, hypercalcemia, and spinal cord compression. These complications can significantly impair a patient's quality of life [1]. Bone-modifying agents (BMAs), including bisphosphonates and denosumab, have been shown to reduce the incidence of SREs in the setting of metastatic cancer [2, 3]. However, there is limited data regarding the optimal timing for initiation, appropriate dosing frequency, and clear criteria for discontinuation. While current guidelines advocate for the aggressive treatment of bone metastases [4], endocrinologists often treat malignancies with a favorable prognoses and excellent long-term survival. Therefore, the treatment strategies used in other cancer types may not be directly applicable to these patient populations.

## Differentiated thyroid cancer

The incidence of differentiated thyroid cancer (DTC) is rising with the majority of cases classified as low-risk and associated with an excellent prognosis [5, 6]. However, survival outcomes are significantly worse in patients with distant metastatic disease [7]. Bone is the second most common site of distant metastases, occurring in approximately 1% to 4% of patients with DTC [8]. Bone metastases are an independent predictor of poor prognosis and metastatic disease in the lungs has a better response rate and higher remission rate when compared to metastatic disease in the bone [8-10]. When bone metastases are present, the 10-year survival rates drop significantly, ranging from 14% to 53% [11, 12]. Older age and larger tumor size are associated with decreased overall survival [13, 14]. Bone metastases are more commonly seen in patients over the age of 65 [8]. This is older than the average age of diagnosis for DTC and may reflect a more aggressive disease course in an older population. Compared to papillary thyroid cancer, patients with follicular thyroid cancers are approximately twice as likely to develop bone metastases and more likely to have an SRE [8]. Despite this, the overall survival is thought to be similar between the histologic subtypes even in the setting of bone metastases [15].

Bone metastases from DTC will typically be lytic lesions. Radioactive iodine (RAI) is used to detect small or symptomatic metastases while [^18^F]-fluorodeoxyglucose positron emission tomography (FDG-PET) and cross-sectional imaging with computed tomography (CT) or magnetic resonance imaging (MRI) can be used to detect RAI-refractory disease. The axial skeleton is the most common site of involvement, particularly the spine and pelvis, making vertebral compression fractures the most common fracture type [16, 17]. These patients represent a high risk population and 34% to 78% of patients will either present with or develop an SRE [8, 17]. Among those who experience an initial SRE, 65% of patients will develop a second SRE [17]. The high rates of SRE in patients with thyroid cancer, even with just 1 or 2 bone metastases, are comparable to the rates seen in breast and prostate cancer [18].

Despite the significant clinical implications, the evidence examining the treatment and outcomes of bone metastases in differentiated thyroid cancer is limited.

### Case # 1

**Figure 1:**
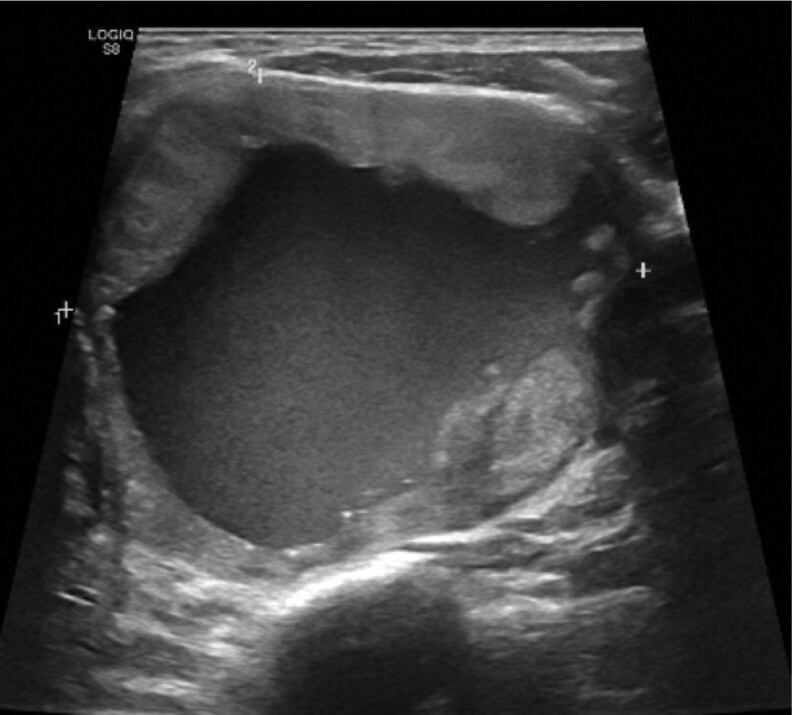
Case #1 preoperative thyroid ultrasound with a 5.9-cm mixed cystic solid left thyroid nodule.

**Figure 2:**
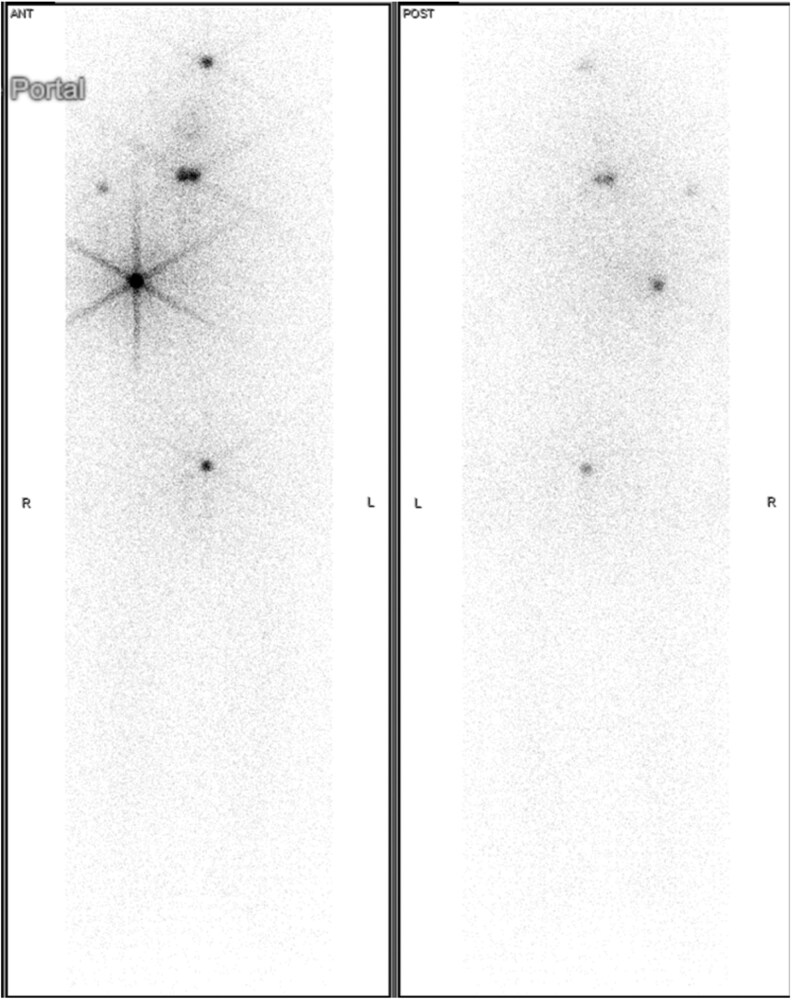
Case #1 posttreatment whole-body scan after first RAI treatment with 72 mCi of ^131^I. In addition to the uptake noted in the thyroid bed, focal ^131^I uptake is noted in the left superior calvarium, right shoulder girdle region, and right anterior hemithorax.

A 51-year-old male with progressive dysphagia symptoms was found to have 5.9-cm mixed cystic solid left thyroid nodule on ultrasound (Fig. 1). The thyroid nodule was biopsied twice with benign pathology (Bethesda II). However, because of continued growth of the thyroid nodule and the patient's compressive symptoms, he elected to proceed with a left thyroid lobectomy. Surgical pathology was notable for a 7.5-cm encapsulated follicular thyroid carcinoma with extensive vascular invasion. Given the extensive vascular invasion, the patient was classified as high risk for recurrence. He underwent a completion right hemithyroidectomy with benign pathology and was treated with a 72 mCi dose of ^131^I using Thyrogen stimulation. The patient's posttherapy whole-body scan showed uptake in the thyroid bed with 3 additional foci of activity in the left superior calvarium, right shoulder girdle region, and right anterior hemithorax (Fig. 2). There was an additional focus of uptake in the central lower pelvis that was thought to be bladder. Stimulated thyroglobulin (TG) level was 188 ng/mL with negative thyroglobulin antibodies. A CT head and CT chest did not identify any structural correlate for the RAI-avid bone lesions. The patient was asymptomatic from his bone disease.

**Figure 3:**
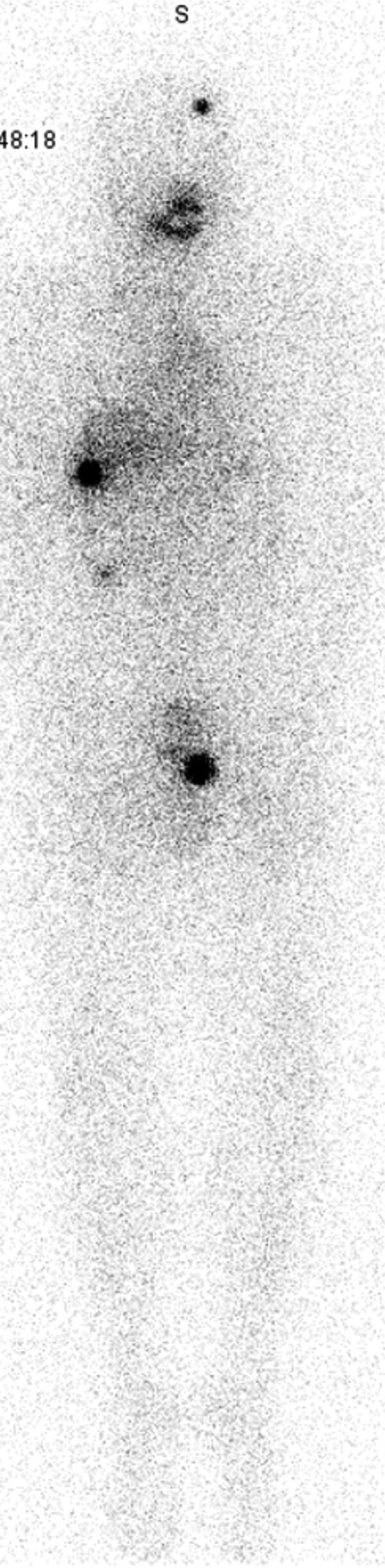
Case #1 posttreatment whole-body scan after second RAI treatment with 152 mCi of ^131^I. Focal ^131^I uptake is noted in the left superior calvarium, right sixth rib, and left superior pubic ramus.

The patient was kept on TSH suppression and did not receive BMAs. The suppressed TG reached a nadir of 3.5 ng/mL. One year after his initial RAI treatment, patient underwent an RAI-whole body scan after thyroid hormone withdrawal. The RAI whole-body scan showed focal uptake in the left superior calvarium and right sixth rib. He was treated with 152 mCi dose of ^131^I. Posttherapy whole-body scan with single-photon emission CT with integrated CT was significant for uptake in the left superior calvarium, right sixth rib, and left superior pubic ramus (Fig. 3). This was consistent with persistent RAI uptake in the osseous metastases that were present on the posttherapy whole-body scan from 1 year prior. The previously seen focal uptake in the right shoulder girdle had resolved. Stimulated TG level was 11.8 ng/mL with negative TG antibodies. The patient continues to be on TSH suppression with close follow up.

### Case #1 discussion: the role of RAI and the treatment of bone metastases without a structural correlate

There are no prospective, randomized clinical studies that have evaluated the role of RAI therapy for the treatment of bone metastases. However, RAI therapy remains an important part of thyroid cancer management and is recommended for iodine-avid bone metastases [6]. RAI avidity and treatment have been consistently associated with improved survival outcomes [6, 10, 16, 19, 20]. In a retrospective study of 444 patients with distant metastatic disease, those who achieved negative imaging studies after RAI treatment, including negative RAI whole-body scan and negative conventional radiographs, had significantly improved overall survival compared to those who did not (92% vs 19%) [10]. When combined with other modalities such as external beam radiation, Cyberknife, systemic therapies, or arterial embolization, RAI treatment is associated with an increase in overall survival compared to patients treated with RAI alone [21]. In about one third of patients with bone metastases from DTC, RAI treatment alone or in conjunction with localized treatment can lead to a complete response in the bone [22]. Unfortunately, approximately 20% of bone metastases lack RAI avidity on the initial RAI whole-body scan, and RAI-refractory bone disease has a worse prognosis [10]. Age is an important prognostic factor, and patients with RAI-avid bone metastases that are younger have higher rates of complete response and better overall survival [12, 20, 23]. Patients with bone metastases diagnosed prior to RAI therapy have a worse prognosis which could be due to a larger tumor burden, more symptomatic disease, or development of SREs at the time of initial diagnosis [15]. RAI has also demonstrated palliative benefits with approximately one third of symptomatic patients experiencing complete relief from bone pain after RAI therapy for iodine-avid bone lesions [24].

Patients with RAI-avid bone metastases without a corresponding structural lesion on imaging appear to represent a distinct subgroup with more favorable clinical outcomes. In these patients, the complete response rate increases significantly from 46% to 82% [12]. Additional studies have shown that these patients rarely develop SREs [17, 25]. This favorable long-term prognosis suggests that either early detection of microscopic disease allows for more effective RAI therapy, or that these lesions represent a less aggressive tumor type. However, long-term mortality data in this population are limited, and it remains unclear whether these patients should be classified as having advanced thyroid cancer.

Case #1 highlights that RAI can be an effective treatment in patients with bone metastases. The patient demonstrated a significant decline in his stimulated TG following the first dose of RAI, consistent with a biochemical response to therapy. Although the patient is older than 45 years old, which was the age cutoff used in earlier studies evaluating age as a prognostic factor, the patient's asymptomatic presentation, the persistent RAI avidity of the bone metastases, and the absence of structural correlates for his RAI-avid bone lesions suggest that he will likely have a favorable prognosis and lower risk of developing SREs.

### Case #2

A 47-year-old male presented with an enlarging palpable right neck mass. Neck ultrasound was notable for a 6.1-cm right neck mass lateral to the thyroid. The patient underwent a biopsy of the right thyroid nodule, which was suspicious for a follicular neoplasm (Bethesda IV); a biopsy of a right neck mass was notable for thyroid parenchyma with macrofollicular architecture and mild nuclear atypia. The patient underwent a total thyroidectomy with bilateral central and lateral neck dissection. Surgical pathology was notable for multifocal follicular-variant papillary thyroid carcinoma with positive margins, lymphatic invasion, and multiple positive lymph nodes in the central and lateral neck compartments with extranodal extension.

**Figure 4:**
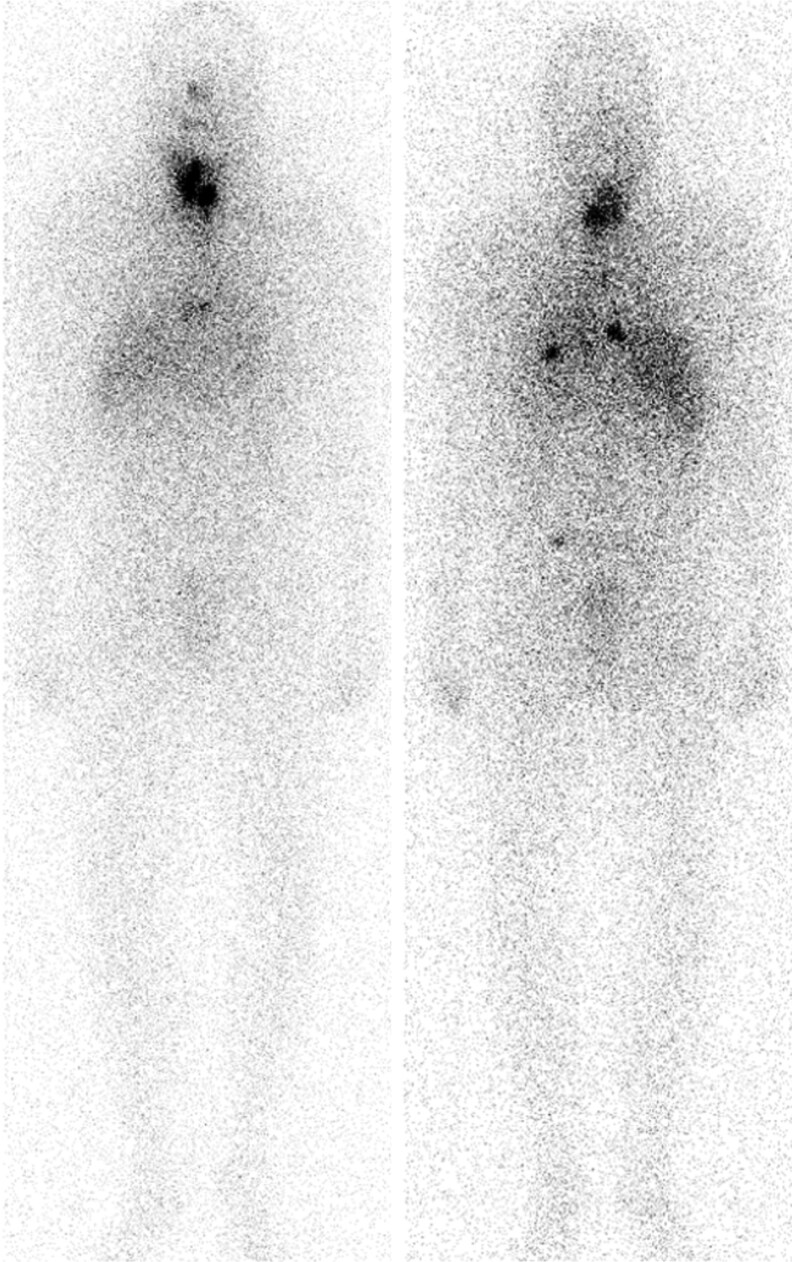
Case #2 posttreatment whole-body scan after treatment with 127 mCi ^131^I. In addition to the uptake noted in the thyroid bed, focal ^131^I uptake is noted in the bilateral iliac bones, left rib, and T8/L1 vertebrae region.

**Figure 5:**
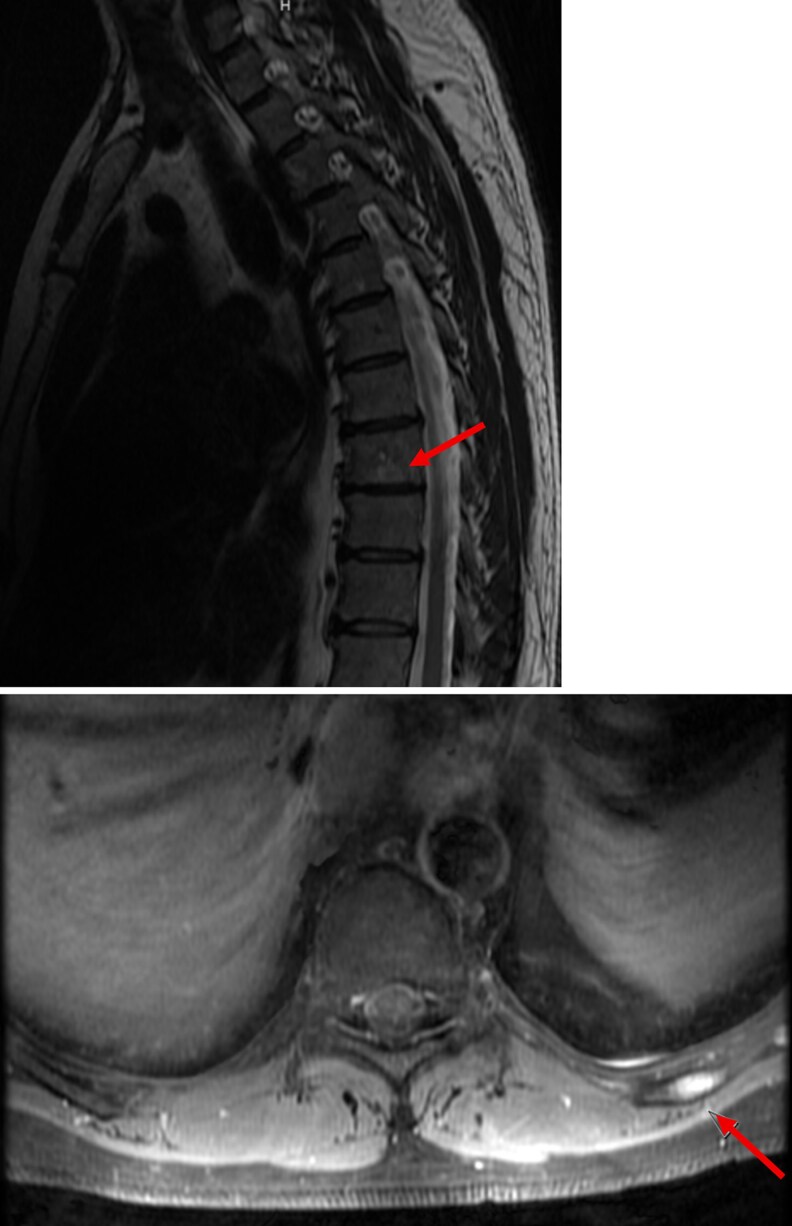
Case #2 MRI thoracic spine. (Top) 10-mm T1 hypointense lesion with mild enhancement in the T8 vertebral body. (Bottom) Left rib with expansile enhancement.

Because the patient was considered high risk for recurrence, he was treated with 127 mCi of ^131^I after thyroid hormone withdrawal. The patient's posttherapy whole-body scan was completed 7 days after treatment and was notable for uptake in the bilateral thyroid bed, bilateral iliac bones, left rib, and the T8/L1 vertebrae region (Fig. 4). Stimulated thyroglobulin level was 999.6 ng/mL with negative thyroglobulin antibodies. An MRI scan of the thoracic spine and pelvis was obtained to evaluate for a structural correlate and establish a baseline for monitoring interval changes in size and treatment response. There were no structural lesions in the pelvis but MRI of the thoracic spine showed a T1 hypointense lesion in the inferior endplate of the T8 vertebral body and an expansile enhancement in the posterior left rib (Fig. 5). Given the presence of bone metastases and concern that small pulmonary metastases may be below the threshold to be seen on an RAI whole body scan, a CT scan of the chest was also obtained and demonstrated bilateral solid pulmonary nodules measuring up to 3 mm, suspicious for pulmonary metastases.

The patient was kept on TSH suppression and his suppressed TG reached a nadir of 14.3 ng/mL 3 months after RAI treatment. The patient had a physically demanding job and was concerned about the risk of SREs related to his bone metastases. After multidisciplinary discussion, given multiple sites of bone metastases including at the spine and hip, patient was started on zoledronic acid 4 mg every 3 months. His TG continued to rise with a doubling time of approximately 6 months. An MRI scan of the thoracic spine obtained about 6 months after RAI treatment showed stable but persistent bone lesion in the T8 vertebral body. A CT of the chest showed new and enlarging pulmonary nodules. One year after RAI treatment, patient had a repeat RAI whole-body scan after thyroid hormone withdrawal. The scan showed uptake in the left 9th rib and T8 vertebral body. The patient was treated with 203mCi ^131^I. A posttherapy whole-body scan conducted 7 days after RAI treatment demonstrated similar uptake in the left 9th rib and T8 vertebral body but also new uptake in the sternum. Stimulated TG level was 916.4 ng/mL with negative TG antibodies. Follow-up MRI scans of the spine continued to show stable disease and repeat RAI whole-body scan was negative. Therefore, zoledronic acid was discontinued after 2 years of treatment.

### Case #2 discussion: the role of BMAs in bone metastases from thyroid cancer

BMAs, such as bisphosphonates or denosumab, have been shown to effectively prevent or delay SREs in patients with solid tumors [26]. Bisphosphonates bind to the bone matrix and are taken up by osteoclasts, ultimately leading to their apoptosis and decreased bone resorption [27]. Denosumab, a monoclonal antibody, functions by binding to receptor activator of nuclear factor kappa-B ligand, thereby inhibiting osteoclast activity and reducing bone resorption [28]. The data on the use of BMAs in thyroid cancer are limited. The optimal timing for initiation, dosing intervals, and treatment duration remains unclear in this patient population.

Studies evaluating the use of BMAs in thyroid cancer are limited and primarily consist of retrospective, single-center studies with small sizes, variable treatment protocols, and heterogenous patient populations (Table 1). Treatment with zoledronic acid has also been shown to significantly delay the onset of skeletal complications, demonstrating greater efficacy than other treatment modalities, including surgery, RAI, or external beam radiation therapy [30]. It may also reduce the incidence and delay the onset of metastatic spinal cord compression, one of the most severe consequences of bone metastases [31]. Although denosumab has been shown to be more effective than zoledronic acid in delaying SREs in patients with solid tumors, patients with thyroid cancer were limited in these studies [33]. Among patients with thyroid cancer who received BMAs for their bone metastases, 75% had already experienced at least 1 clinical SRE at the time of treatment initiation [34]. Despite the high incidence of SREs in patients with bone metastases from thyroid cancer, treatment rates remain low, with only 22.4% of patients being treated [34]. This is a potential missed opportunity for earlier intervention. Further research is needed to identify patients at the highest risk for SREs who would derive the greatest benefits from BMA treatment.

**Table 1:** Impact of bone-modifying agents in patients with bone metastases from thyroid cancer

Author (year)	Study design	Patient characteristics	Medication, dose, frequency, duration	Bone-related outcomes
Vitale et al (2001) [29]	Prospective study; single-center	10 patients; 6 follicular, 2 papillary, 2 medullary	Pamidronate, 90 mg, monthly. 12 cycles (average 11.5 cycles)	Decrease in bone pain (*P* = .0052). Improved performance status (*P* = .051). Partial radiographic response in 20% of patients.
Orita et al (2011) [30]	Retrospective study; single-center	50 patients; 24 follicular, 26 papillary	Zoledronic acid, 4 mg, monthly. Average 16 cycles (range 1-33)	Decrease in SRE (*P* = .007). Delay in time to first SRE (*P* = .04).
Orita et al (2015) [31]	Prospective study; single-center	19 patients; 5 follicular, 14 papillary	Zoledronic acid, 4 mg, monthly. Average 14 cycles (range 3-31)	42% of patients had 1 SRE during the observation period. Significant reduction of MSCC (*P* = .017) and delay onset of MSCC (*P* = .042). Decrease in pain scores in 5 patients and aggravation in 6 patients.
Andrade et al (2019) [32]	Retrospective study; single-center	50 patients; 18 follicular, 25 papillary, 7 poorly differentiated	Zoledronic acid, 4 mg, monthly. Median 7.5 cycles (range 1-25)	Decrease in SRE (*P* = .006), Increase in overall survival (*P* = .06).
Wu et al (2019) [21]	Retrospective study; single-center	77 patients; 42 follicular, 35 papillary	30 patients (39%) received bisphosphonate (unknown dose). 22 patients (29%) received Denosumab (unknown dose).	Improved survival for patients who received bisphosphonates (7.8 vs 6.0 years; *P* = .31) and denosumab (7.7 vs 5.2 years; *P* = .03)

Abbreviations: MSCC, metastatic spinal cord compression; SRE, skeletal-related event.

Bone-directed treatment can also decrease bone pain and improve quality of life. In a prospective study of 10 patients with thyroid cancer that had undergone extensive treatment with surgery, RAI, external radiotherapy, and chemotherapy but continued to have painful bone metastases, pamidronate was shown to significantly reduce bone pain (*P* = .0052) and improve performance status (*P* = .051) [29]. Zoledronic acid has also been show to decrease pain scores and reduce analgesic agents in almost one third of patients with bone metastases from DTC [31].

The optimal treatment dosing frequency of BMAs in patients with DTC remains unclear. Although most thyroid cancer studies have used zoledronic acid at 4 mg every 4 weeks, emerging evidence suggests that extending the dosing interval to every 12 weeks can still be effective and safe. This approach has been extrapolated from studies in breast cancer, prostate cancer, and multiple myeloma, which have shown that a 12-week doing schedule does not increase SREs but may decrease treatment-related toxicities [35, 36]. The American Thyroid Association has endorsed the 12-week dosing schedule, although it acknowledges there are limited data within thyroid cancer supporting this recommendation [6]. Larger, prospective trials are needed to better determine the optimal dosing strategies and schedule.

The selection of BMA should be guided by individual patient factors including risk of SRE, status of overall tumor control, cost, and patient preference. Denosumab may be more effective at reducing SREs and can be used in patients with reduced renal function. However, abrupt cessation of denosumab is associated with rapid rebound bone loss and risk of multiple vertebral fractures [37, 38], and dosing regimens other than monthly administration have not been tested. Intravenous bisphosphonate may be more cost effective, are not associated with rebound bone loss on cessation and can be administered either monthly or every 3 months.

Although BMAs offer clear benefits in reducing SREs and alleviating bone pain, their long-term use needs to be carefully considered in patients with thyroid cancer who can have long life expectancies, even in the setting of metastatic disease. With prolonged antiresorptive therapy, patients are at risk for rare side effects including osteonecrosis of the jaw (ONJ) and atypical femoral fractures (AFF). ONJ is rare but the risk is dose-dependent and the majority of cases been reported in oncology patients [39]. Although the incidence of ONJ in patients with thyroid cancer is poorly defined, the reported rates are alarmingly high. In small retrospective studies, ONJ occurred in up to 9% of patients treated with monthly zoledronic acid and in up to 26% of patients treated with denosumab [30, 40]. These rates are substantially higher than the 1.7% incidence observed in a meta-analysis of randomized controlled trials, which primarily included patients with breast cancer, prostate cancer, and myeloma [41]. Patients with thyroid cancer may be at a higher risk for ONJ if patients receive external beam radiation therapy to the neck, which can be used in patients with advanced disease. Furthermore, the use of tyrosine kinase inhibitors as systemic therapy can further elevate this risk [42, 43]. Given these considerations, the frequency and duration of BMAs in patients with advanced thyroid cancer should be guided by a multidisciplinary team. Therapy should be tailored to the patient's individual risk profile to maximize therapeutic benefit but minimize complications.

Adjuvant bisphosphonate use has been associated with improved overall and disease-specific survival in postmenopausal women with early breast cancer [44]. However, the potential impact of BMAs on survival outcomes in DTC has not been seen. Retrospective studies have suggested a trend toward improved overall survival in patients treated with zoledronic acid, and a longer median survival in patients receiving denosumab [21, 32]. These findings are based on small patient cohorts, and multivariate analysis has not identified use of BMAs as an independent prognostic factor for improved survival [34]. Larger, prospective studies are needed but there is currently insufficient evidence to suggest a direct survival benefit from BMA therapy in this population.

Bone-modifying agents reduce skeletal-related events and bone pain in patients with thyroid cancer and bone metastases. However, the available data are limited to small, retrospective studies and their use in patients with thyroid cancer is largely underexplored. Critical questions remain regarding optimal timing, dosing schedule, and long-term safety. Further prospective studies are needed to clarify how to best optimize the use of BMAs in thyroid cancer to guide evidence-based recommendations.

Case #2 highlights the complexities involved in deciding whether to initiate BMAs, as well as the importance of multidisciplinary care and shared decision-making. There is insufficient evidence that adjuvant BMAs use improves overall or disease specific survival in patients with thyroid cancer. In this patient, the TG level continued to rise despite initiation of zoledronic acid, which was likely driven by progression of nonosseous disease. This case also underscores the importance of de-escalation therapy, especially when bone disease remains stable and is not progressing.

## Neuroendocrine tumors

### Case #3

**Figure 6:**
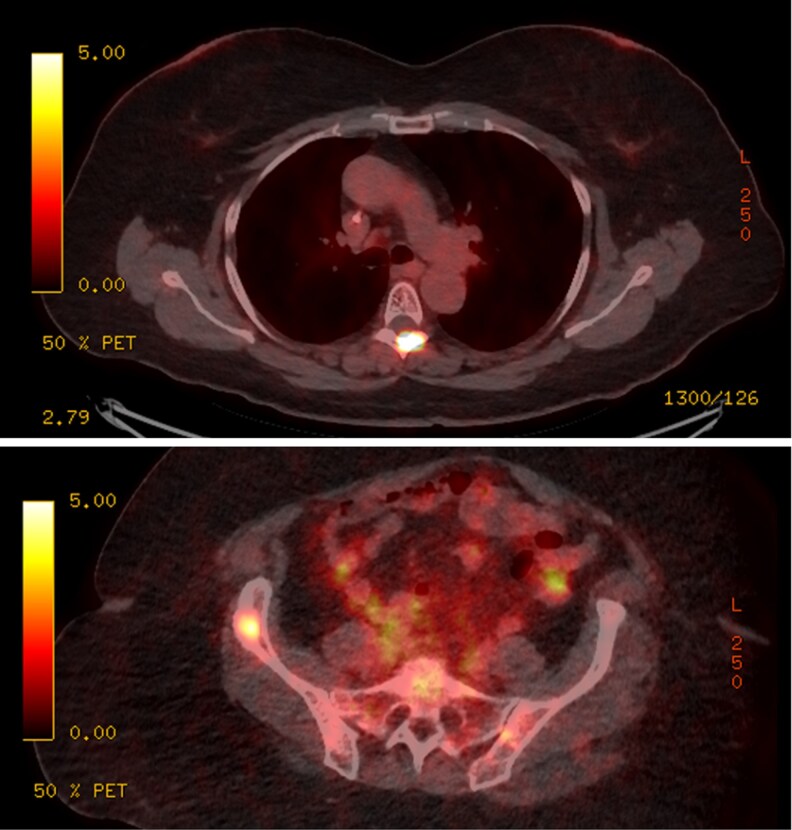
Case #3 ^68^Ga-DOTATATE PET/CT with intense radiotracer activity in (top) the left lamina of T5 vertebra and (bottom) anterior right iliac.

A 64-year-old female underwent wedge resection of the right middle lobe of the lung for a 3.4-cm pleural-based mass. Pathology revealed intermediate-grade neuroendocrine tumor. She received 6 cycles of carboplatin/etoposide and 50 Gy of external beam radiation therapy. MRI of the abdomen revealed hepatic masses, core needle biopsy confirmed a metastatic neuroendocrine tumor (NET), and she was started on everolimus and monthly octreotide and underwent transarterial embolization of the liver. No bone lesions were noted on an octreotide scan nor on serial chest CT and abdominal MRI scans over the next year. Twenty-two months after her surgical resection, a ^68^Ga-DOTATATE PET scan revealed 3 hypermetabolic foci in the liver and numerous foci in the bones, including the left anterior first rib, posterior element of T5, 8th rib, L2, right anterior iliac wing, and right inferior sacrum (Fig. 6). She was started on zoledronic acid 4 mg every 3 months.

### Case #3 discussion: the role of BMAs in bone metastases from neuroendocrine tumors

Neuroendocrine tumors arise from neuroendocrine cells, most often in the gastrointestinal or respiratory tracts. Bone metastases have been reported in 4% to 20% of patients with a NET [45-54]. Bone metastases in NETs most commonly arise from primary tumors in the small intestine, pancreas, and lung [47, 48, 50, 55], and are synchronous (diagnosed at the same time as the primary tumor) in approximately one third to one half of cases [45, 48, 50, 52, 53]. With the introduction of ^68^Ga-DOTA-peptide PET/CT scans, the incidence has increased because of improved detection [56, 57]. In a small autopsy study, bone metastases were noted in 42% of subjects with carcinoid tumors, suggesting that bone metastases may still be underdetected [58].

More than half of patients with NET bone metastases are symptomatic, with pain as the most common symptom, as well as other skeletal-related events including spinal cord compression, fracture, and hypercalcemia [45, 48, 52, 57]. Median overall survival is significantly shorter with bone metastases (40-60 months), compared to 84 to 100 months without bone metastases [45, 46, 48, 52, 56].

Management options for NET bone metastases include chemotherapy, peptide receptor radionuclide therapy, external beam radiation, surgery, and BMAs [59]. Despite the risk of SREs, only 25% to 50% of patients with NET bone metastases are treated with bisphosphonates or denosumab [45, 46, 52, 53]. There are no randomized controlled trials or prospective studies of BMAs in NET bone metastases.

As with differentiated thyroid cancer, the optimal timing, dosage, and duration of BMA therapy remains to be determined in NET bone metastases. The increased identification of bone metastases by ^68^Ga-DOTA-peptide PET/CT scans suggests that bone metastases previously went unrecognized and therefore may be asymptomatic for some time. However, because SREs are common in NET bone metastases, it is reasonable to initiate BMA at the time of diagnosis.

There are no studies investigating the optimal duration of BMA therapy. The duration of follow-up in studies demonstrating the efficacy of BMAs in reducing SREs is typically less than 3 years. In patients with NETs, even with bone metastases, median survival may exceed 3 to 5 years [45, 46, 48, 52, 56]. Because bisphosphonates have a prolonged half-life in bone, our current practice is to initiate IV zoledronic acid every 3 months in patients with newly identified bone metastases. If imaging studies reveal stability of bone metastases over 1 to 2 years, after discussion with the patient and the oncology team, we will consider reducing the frequency to every 6 months, and if stable for another year or 2, even to every 12 months. At any time, if imaging studies reveal progression of bone lesions the dosing frequency can be increased back to every 3 months.

Because prolonged exposure to bisphosphonates increases the risk of ONJ and AFF, in patients treated with bisphosphonates for osteoporosis, consideration of a drug holiday is recommended after 5 years on oral bisphosphonates or 3 years on IV bisphosphonates for individuals not at high risk of fracture [60]. The risk of atypical fracture is rapidly reduced on cessation of bisphosphonates [61]. The role of drug holidays in the management of bone metastases is unknown. Because bone metastases are incurable, guidelines have generally recommended indefinite use of BMAs until no longer consistent with goals of care [4, 62], However, in a patient with stable bone disease treated for several years with bisphosphonates, it may be reasonable to consider a drug holiday after discussion of risk and benefits.

It is currently unknown whether adjuvant bisphosphonate therapy extends overall survival in patients with NETs [63]. To date. retrospective studies have not demonstrated improved overall survival with bisphosphonates [46, 48].

Case #3 highlights the changing landscape of the management of NET with bone metastases, as newer imaging modalities have revealed that bone metastases may be more common than previously appreciated. Although bone metastases are associated with increased SREs and reduced overall survival, the optimal timing, dose, and duration of BMA therapy in patients with bone metastases from NET remains uncertain. With median survival measured in years, again the importance of multidisciplinary care and shared decision-making must be emphasized to maximize benefit while minimizing risk.

## Conclusion

The management of bone metastases in patients with DTC and NETs remains a significant clinical challenge because of the lack of prospective, disease-specific studies to guide management. Although these patients are at risk for SREs that significantly impact morbidity and quality of life, optimal strategies for prevention and management are understudied. Current recommendations remain extrapolated from more aggressive cancers and there is a need for research in these more indolent cancers.

Targeted imaging and treatment, including RAI and ^68^Ga-DOTA-peptide PET/CT scans, have improved the detection and prognosis in patients with bone metastases. BMAs, such as bisphosphonates and denosumab, have been shown to reduce SREs, prolong the time until a subsequent SRE, and decrease pain. Despite these benefits, BMAs have not made a significant impact on overall survival. However, further studies are still needed to determine the optimal timing for initiation of BMAs, the most effective dosing interval, and the overall duration of therapy. It is currently unknown whether treatment should be continued indefinitely in patients with bone metastases from DTC or NETS but with stable bone disease. In addition, in patients anticipated to have a long life expectancy, the risks of treatment-related complications such as ONJ and AFF need to be considered.

A multidisciplinary, individualized approach is essential to optimize outcomes and balance efficacy with safety. Future prospective studies focused on DTC and NETs are needed to establish evidence-based guidelines to improve long-terms outcomes and quality of life for these patients.

## Data Availability

Data sharing is not applicable to this article as no datasets were generated or analyzed during the current study.

## Abbreviations

AFF: atypical femoral fracture
BMA: bone-modifying agent
CT: computed tomography
DTC: differentiated thyroid cancer
MRI: magnetic resonance imaging
NET: neuroendocrine tumor
ONJ: osteonecrosis of the jaw
RAI: radioactive iodine
SRE: skeletal-related event
TG: thyroglobulin
